# A Systematic Study of Bovine Viral Diarrhoea Virus Co-Infection with Other Pathogens

**DOI:** 10.3390/v17050700

**Published:** 2025-05-14

**Authors:** Zhiwei Hou, Jiahui Wang, Bin Tan, Shuqin Zhang

**Affiliations:** Institute of Special Animal and Plant Sciences, Chinese Academy of Agricultural Sciences, No. 4899 Juye Street, Changchun 130112, China; 17331015213@163.com (Z.H.); x18545780919@163.com (J.W.); tanbin@caas.cn (B.T.)

**Keywords:** bovine viral diarrhoea virus, co-infection, synergy, pathogens

## Abstract

Bovine viral diarrhoea virus (BVDV) is the causative agent of bovine viral diarrhoea/mucocutaneous disease (BVD-MD). Its associated co-infections pose a threat to the cattle industry, which is becoming a key breakthrough in the global system of prevention in the cattle industry. In recent years, cases of co-infection have occurred and been reported from time to time, and this situation not only poses certain difficulties in controlling the outbreak and in treatment in the farming industry, but also poses considerable challenges in detection and diagnosis. In this review, by systematically integrating studies on BVDV co-infection, we firstly compared and analysed the characteristics of BVDV co-infection with viruses, bacteria and other pathogens in in vivo/in vitro models in terms of synergism, host immune response and epidemiological transmission. Then we systematically constructed a BVDV Co-infection Impact Map, which demonstrates a paradigm of pathogen–host–immune interactions in the transmission of BVDV and provides a theoretical framework for breaking through the current precision diagnostic strategies and showcasing the effectiveness of integrated prevention and control.

## 1. Background

Bovine viral diarrhoea virus (BVDV) belongs to the Flaviviridae family of viral particles of the genus Pestivirus, which includes three bovine viruses, BVDV-1 (Pestivirus bovis), BVDV-2 (Pestivirus tauri) and HoBi-like pestivirus (Pestivirus brazilense) [1,2]. The BVDV genome is a single-stranded positive RNA approximately 12.3 kb in size, with an open reading frame (ORF) that can encode a single major protein of 449 kDa [3,4]. The viral particles are spherical to hemispherical [5]. Its protein shell contains an E1-E2 heterodimer, which enters cells by binding to bovine CD46 molecules [6,7]. BVDV not only infects cattle but also crosses species to infect a wide range of animals, e.g., sheep and pigs [8,9,10]. BVDV infections are present in many parts of the world, and acute infections are always accompanied by circulating lymphopenia and immunosuppression [11,12], resulting in a lowered resistance of the affected animal during which it is very susceptible to secondary infections and highly vulnerable to the pathogen [13,14]. Depending on the strain of infection, host condition and secondary infections, infected animals develop different degrees of intestinal, respiratory and reproductive diseases [15,16], with non-cytopathic infections predominating. When persistent non-cytopathic BVDV infections are combined with cytopathic BVDV infections, mucosal disease (M-D) may result [10,17,18,19,20].

BVD cases were first reported in the United States in 1946 [21,22], but this does not imply a first appearance. Evolutionary analyses have postulated that the ancestor of BVDV appeared around 1629–1736. It is postulated that BVDV-1 first diverged from BVDV-2 around 1743 and that it became progressively more diversified around 1803, more than a century before it was first reported [23]. BVDV-1 infections can present with clinical symptoms of varying severity depending on the genotype of the infecting virus, typically presenting with depression, fever, conjunctivitis, runny nose, cough and diarrhoea [24]. In animals infected with different genotypes of BVDV-2, the severity and duration of clinical symptoms will also vary, often causing gastrointestinal symptoms accompanied by a certain degree of watery diarrhoea and mental depression [25]. Infection of pregnant females by BVDV can also lead to various reproductive disorders, such as infertility, abortion and weak calves [15]. There are cases in nature where BVDV-1 and BVDV-2 co-infected the same animal [26].

Mixed infections involving BVDV are generally more serious than simple infections caused by BVDV, and the occurrence of bovine respiratory disease complex (BRDC) often results in large economic losses for the cattle farming industry, where the viral pathogens associated with BRDC include the following: bovine herpesvirus type 1 (BHV-1), parainfluenza virus (PI-3V), bovine viral diarrhoea virus (BVDV) and bovine respiratory syncytial virus (BRSV), among others. Bacterial pathogens associated with BRDC include the following: *Mannheimia haemolytica*, *Mycoplasma bovis*, *Pasteurella multocida* and *Histophilus somnus* [27,28,29]. Although BVDV is not the virus primarily responsible for the respiratory disease associated with it, the synergistic effect of BVDV-induced immunosuppression or co-infection can enhance the pathogenicity of the pathogen [30]. Moreover, BVDV often co-infects with various pathogens (BRSV, BCoV, P. multocida) [31]. Viruses that co-infect with BVDV include BHV, PI-3V, BRSV, BoCV, BRV, Orbivirus, PEDV, etc. Bacteria that commonly co-infect with BVDV include *Mannheimia haemolytica*, Salmonella and Mycobacterium bovis. Even co-infections with other pathogens, such as *Mycoplasma bovis* and *Neospora caninum*, occur from time to time. Moreover, BVDV mixed infections are not limited to ruminants such as cattle; there are many cases of mixed infections with BVDV in swine, with pathogens such as classical swine fever virus, porcine epidemic diarrhoea virus, porcine circovirus 2, etc. [32,33,34,35], and cases of mixed infections with multiple pathogens (BVDV, PDCOV, PKoV, PASTV, TGEV) have also occurred [34]. It is noteworthy that most people still know more about the relationship between single pathogens and their hosts and the impact it has, while the relationship between BVDV-associated multi-pathogen co-infections and their impacts are not yet very systematically understood. In-depth understanding of the presence of cases of pathogen co-infection, in vivo or in vitro experiments of host or cellular responses and the dissection of the mechanisms involved in the emergence of synergistic or antagonistic interactions following pathogen co-infections and their impact on the organism are essential for our diagnosis, infectious disease assessment and development of strategies to reduce the incidence of infectious diseases and losses. Therefore, this paper systematically discusses the interactions between BVDV and a variety of pathogens and their effects on the host, with the aim of providing assistance in the accurate diagnosis of co-infections, disease prevention and control, and vaccine use and production.

## 2. Co-Infection of BVDV with Viruses

Co-infections are less well studied than single virus infections, and the mechanisms of co-infections are more complex, requiring consideration of virus–virus and virus–organism interrelationships and effects. The role of co-infections is also more important in this field, so here we summarise and analyse the occurrence and interactions of a limited number of cases and experiments of co-infections of BVDV with other viruses in order to better understand the impact of viral co-infections (Table 1).

### 2.1. Co-Infection of BVDV with BHV

Bovine herpesvirus (BHV), also known as ruminant herpesvirus, belongs to the Herpesviridae family and is a spherical viral particle with an enveloped linear double-stranded DNA genome [58], currently classified as Bovine herpesvirus 1 (BHV-1), Bovine herpesvirus 2 (BHV-2), Bovine herpesvirus 4 (BHV-4), Bovine herpesvirus 5 (BHV-5), and Bovine herpesvirus 6 (BHV-6) [59].

BHV-1 can cause different clinical signs such as infectious bovine rhinotracheitis (IBR), infectious pustular vaginitis (IPV), and infectious pustular bullous impetigo (IPB) [60], and latently infected animals can be activated by a number of stimuli to excrete the virus [61], which can be considered as a lifelong infection [62,63]. Moreover, BVDV-positive animals are associated with increased BHV-1 seropositivity and herd size [64]. Where the risk of BHV-1 positivity was also associated with BVDV serodiagnostic status, age, sex, year of sampling, herd type and herd size [65]. During virus isolation from lung tissue of a group of feeder calves with acute respiratory disease [66], BVDV and BHV-1 were isolated from one of the lungs, BVDV and BHV-1 were successively isolated from another group of purchased calves [67], and a group of 30 unvaccinated cows showed mixed infections of the two viruses [68], and the presence of mixed infections of BHV-1 has also been detected by using microarray technology [69], suggesting that the two viruses can be co-infected under natural conditions and that co-infections of the two viruses are very likely to occur [70,71,72,73], often in combination with a variety of other pathogens [74]. Co-infections of BVDV with BHV result in a more severe clinical response [44,46], with BVDV inhibiting the proliferation of and response to CD8^+^ and CD4^+^ lymphocytes [44,75]. Infection with BVDV followed by BHV-1 resulted in pathological changes of thymic atrophy, which affected the number of T-cells and their mediated immune responses, and exacerbated immunosuppression to a certain extent, whereas animals infected with BHV-1 alone did not show this lesion [38].

In addition, in studies on thymic immune cells, it was shown that BHV-1 also seems to induce some immune alterations [39]. In another bovine outbreak investigation and study, it was shown that BVDV-induced immunosuppression predisposes animals to secondary infection with BHV-1 [45] and enhances the pathogenic effects of BHV-1 in cattle [36], which may be related to increased secretion of pro-inflammatory cytokines (TNF-α) and decreased production of anti-inflammatory cytokines (IL-10) following secondary infection [40], resulting in the promotion of inflammation in the lungs and the establishment of a pro-coagulant environment, further disrupting lung homeostasis. The establishment of a procoagulant environment in the lungs further disrupts lung homeostasis, contributing to the earlier development of more severe inflammatory lesions [41], and to a certain extent reduces the calf’s ability to immunologically clear BHV-1 from the lungs [37]. This would promote further multiplication and systemic spread of BHV-1, while the presence of BHV-1 would also be more conducive to the persistence of BVDV in target organs such as the gastrointestinal tract, suggesting some synergy between the two viral parties [42]. The same BHV-1 virus will appear to spread widely in most tissues of BVDV-infected calves, and it is worth noting that BVDV will also usually contain BHV-1 to localised sites of infection, resulting in high localised concentrations of virus, which synergises with BVDV’s ability to reduce BHV-1 clearance from the lungs [37], further prolonging the persistence of the expelled virus, suggesting that in BVDV-infected calves BHV-1 would exhibit enhanced pathogenic and infectious properties [44].

Compared to BHV-1, BHV-4 generally causes low-grade systemic disease [76], but it is also immunosuppressive and often co-infects with other pathogens [77,78]. Clinical manifestations associated with BHV-4 include postpartum uteritis, abortion, and mastitis [79,80]. In a group of 25 animals in which BHV-4 was detected, 14 cases were co-infected with BVDV [81]. In co-infections with BVDV, although BVDV affects BHV-4 replication, it delays expression of the BHV-4 gene and reduces viral load in the extracellular milieu [47], where a more severe clinical response often occurs [46], a phenomenon that suggests that there may be a viral load-independent pathological mechanism (e.g., immune imbalance). It is worth noting that an NCP strain of BVDV-2 was once isolated from a batch of live IBR vaccine and was somewhat infectious [82], which also reminds the vaccine production process to be aware of the risk of contamination of cells or animals used for production that may exacerbate viral transmission; after all, defence against BVDV and BHV-1 is critical for cattle production [83].

### 2.2. Co-Infection of BVDV with PI-3V

PI-3V is a non-segmented negative-stranded RNA enveloped virus belonging to the genus Respiratory Virus in the family Paramyxoviridae [84,85]. PI-3V is spherical to polymorphic, with viral particles consisting of a nucleocapsid surrounded by a lipid envelope and ranging in size from 150 to 200 nm, with a single-stranded genome size of approximately 15 kb, including 6 genes encoding 9 proteins [86]. PI-3V is one of the main pathogens of the bovine respiratory disease complex, which was first isolated in 1959 [87], and infected animals show mild symptoms such as fever, dry cough, and runny nose [86,88]. Although it has been suggested that the prevalence of the disease is not associated with sex [89], some studies have found that females have a higher prevalence than males [90], and the association between the infection and sex is somewhat controversial. Notably, there was also a correlation between prevalence and body weight in similar age groups [91].

In the 460 unvaccinated adult cattle tested, the pathogens BVDV and PI-3V were found to be more common and co-infected at a rate of 19.1% [68]. Similarly, in another group of 979 unvaccinated cattle with the relevant vaccine, the number of BVDV and PI-3 co-infections alone was 521 [92]. A number of cases suggest the possibility of co-infection in farmed herds where both are naturally bred to be co-infected [73,93], and co-infection with both or multiple pathogens is frequent [35,66]. In particular, co-infection of both with BVDV and PI-3V with BHV-1 is also a cause of enteropneumonic disease. It is worth noting that persistently infected calves with BVDV that have normal clinical and immunological performance are able to produce PI-3V-neutralising antibodies when exposed to PI-3V [94], but the BVDV that causes abnormalities in immune function still leaves the animals susceptible to secondary infection with PI-3V [45]. Furthermore, in studies of calves co-infected with BVDV and PI-3V, infection with PI-3 viruses did not seem to significantly alter the pathogenesis of experimentally induced BVDV infection [46], suggesting that the two may cause disease by independent pathways. In an investigational study, prevention and control of outbreaks were not completely reliable with BVDV and PI-3V vaccines alone [95], suggesting that we need to pay attention not only to vaccination but also to preparation in prevention and control management plans.

### 2.3. Co-Infection of BVDV with BCoV

Coronaviruses (CoVs) are large, enveloped, single-stranded positive-sense RNA viruses with a genome of 26 to 32 kb, belonging to the Coronaviridae family, which can infect a wide range of animals and cause respiratory, gastrointestinal and neurological diseases [96,97,98]. Among them, bovine coronavirus (BCoV) is a virus associated with lung and intestinal lesions [97]. BCoV particles are polymorphic and enveloped, with five main structural proteins and a diameter of 65–210 nm, which can cause lesions in the respiratory and intestinal tracts of cattle and other ruminants [99,100]. Depending on the route of infection, it can also affect the respiratory and intestinal stages of infection [101], and can spread rapidly if there is an infected animal in the same unit [55] and it is often used as one of the causes of the BRDC.

Co-infections with multiple pathogens such as BVDV and BCoV occur [31], and there are some significant associations between BVDV and BCoV [102], but the window of enhanced pathogenicity differs between the two viruses [54]. In a single BVDV infection, virus secretion in the nasal cavity is limited, and in rare cases BVDV can be isolated from it, whereas when co-infection with BCoV occurs, BVDV can be isolated from nasal swabs, suggesting that co-infection with BCoV enhances the level of BVDV excretion from the nasal cavity, which can enhance the rate of virus transmission and the extent of infection. At the same time, BCoV may have an impact on the innate immunity of the respiratory tract, and co-infection with BVDV not only creates better conditions for secondary infections, but also synergistically enhances damage to the respiratory tract [54]. It was analysed and deduced from the study of naturally mixed-infected calves that BVDV may also regulate BCoV infection, thus prolonging the duration of virus expulsion [101]. Compared with calves infected with BCoV alone, mixed-infected calves showed lower fecal BCoV loads, which may be due to the direct or indirect interference of BVDV with the replication of BCoV [55]. The interacting mechanisms of such co-infections (synergistic enhancement of transmission, immunosuppression, and pathogenicity) pose a great challenge to the prevention and control of epidemics.

### 2.4. Co-Infection of BVDV with BRSV

Bovine respiratory syncytial virus (BRSV), first isolated and reported in 1970 [103], is an enveloped, non-segmented, negative-stranded RNA virus with a genome size of about 15 kB that translates 11 viral proteins and belongs to the paramyxovirus family, which can be transmitted by aerosol and direct contact [104,105]. The virus mainly replicates in the cells of the respiratory tract and lungs, causing clinical signs such as fever, cough, nasal and ocular secretions [105,106], and is susceptible to secondary bacterial infections during infection, leading to pneumonia, which is one of the main causes of BRDC [107].

Infection with pathogens such as BVDV and BRSV was more common in unvaccinated adult cattle, with co-infection with both BVDV and BRSV detected in 39 of them [68]. In another test of serum samples from 494 unvaccinated cattle, 271 samples containing neutralising antibodies to BVDV and BRSV were detected. In addition, mixed infections with multiple pathogens such as BVDV and BRSV occur [31,66,74].

Co-infected calves show more severe clinical signs, leukopenia and more severe respiratory, pulmonary and intestinal pathology than those infected with any of the viruses alone, and the virus is excreted from the nasal cavity in higher concentrations and for a longer period of time, and there is a certain synergism between the two viruses, which enhances pathogenicity on the organism [51]. When co-infection occurs in a sequential manner, BVDV induces a serum interferon response that inhibits BRSV replication. At the same time, BVDV causes a certain degree of immunosuppression (e.g., a decrease in MHC-II and CD8^+^ lymphocytes), which leads to a delay in the body’s immune response to BRSV, thus increasing the window of transmission for the outward expulsion of BRSV, and relatively lengthening the duration of the transmission of BRSV [52]. This results in a vicious circle of “immune imbalance-virus persistence”. In an in vitro co-infection assay, BVDV2-wt reduced mRNA expression of IRF-3 pathway signalling (the core of interferon regulation) in co-infected cells, weakened host antiviral responses, and enhanced BRSV replication, which better illustrates the relationship between BVDV2-wt and increased BRSV pathogenicity in co-infected animals [49]. In another in vitro study, co-infection with both viruses synergistically inhibited the expression of Fc receptors in bovine alveolar macrophages, which also increased PGE-2 production or inhibited phagocytosis leading to inhibition of phagolysosome-lysosome fusion, and synergistically inhibited O_2_^-^ production and chemokine secretion [53], which resulted in a complete breakdown of immune defences. Co-infection with the two viruses reduced the levels of MHC-II-antigen-expressing cells, such as the spleen, and showed a synergistic inhibition of BoCD8^+^ lymphocytes in the thymus, suggesting that co-infection exacerbates the effects of the co-infection on the lymphatic system of the diseased animals [50]. Overall, these may contribute to the tendency for respiratory and gastrointestinal diseases to become more severe.

### 2.5. Co-Infection of BVDV with BRV

Bovine rotavirus (BRV) is caused by rotaviruses that cause viral diarrhoeal disease. Rotaviruses are enveloped double-stranded RNA viruses with particles of 70–75 nm in size, belonging to the family Eutheroviridae and the genus Rotavirus. Its genome is a fragmented double-stranded RNA, 16–21 kb in size, consisting of 11 fragments. The virus has a three-layered protein capsid consisting of an outer capsid, an inner capsid, and a core capsid [108,109,110]. BRV causes high morbidity and moderate mortality of infections, and infected calves show signs of watery diarrhoea, depression and loss of appetite [111,112].

Cases of co-infection with multiple pathogens such as BVDV and BRV occur [113,114]. Co-infection with BVDV and BRV can be synergistic in the gut compared to calves infected with either BVDV or BRV alone, and the immunosuppressive effects caused by BVDV may enhance the effects of BRV infection, leading to more severe intestinal damage and disease, with lesions such as villous atrophy and submucosal inflammation, suggesting that there is a pathological superimposition effect between the viruses. A significant increase in BVDV titres was seen in ileal tissue during coinfection with BRV, suggesting that BRV also enhances BVDV replication during coinfection [56]. The latter, together with immunosuppression and destruction of the intestinal microenvironment, formed a bidirectional enhancement cycle that would further exacerbate the infection.

### 2.6. Co-Infection of BVDV with Other Ruminant-Associated Viruses

Cyclic viruses are complex, enveloped RNA-free viruses with seven structural proteins and an RNA genome consisting of 10 double-stranded RNA fragments of different sizes, belonging to the family Eutheroviridae [115]. Compared with experiments in bovine testicular cells without non-cytopathogenic (NCP) bovine viral diarrhoea virus infection, NCP BVDV-infected bovine testicular cells showed a cytopathogenic effect (CPE) after overlapping infections with multiple circoviruses and enhanced replication of the circoviruses, which may be related to the END-positive phenomenon in which NCP BVDV inhibited interferon production in BT cells [57,116].

In addition, bosavirus is a previously uncharacterised fine virus, and based on pairwise NS1 comparisons, bosavirus was proposed as a genus Copiparvovirus [117]. Although the aetiological significance of bosavirus infections and the effects of co-infection are unknown, it is important to note that cases of co-infection of BVDV and bosaviruses exist, and that both viruses can persistently infect bison [118].

Cases of co-infection with other pathogens have also occurred with combinations of BVDV and BPV, BVDV and PPRV, BVDV and PCPV, BVDV and BLV, BVDV and BAV, and BVDV and FMDV [113,119,120,121,122,123]. Experimental data have also shown that co-infection with BVDV and BAV can lead to more complex seroconversion and clinical symptoms [36]. These co-infection cases and experiments are very rare, but we need to be aware of this aspect.

### 2.7. Co-Infections Associated with BVDV and Porcine Viruses

Although BVDV is primarily a pathogen that infects cattle, other animals such as pigs can also be infected [33]. This suggests that we need to be aware of the dangers and impacts caused by BVDV infections not only in ruminant farming such as cattle, but also in the farming of animals such as pigs (Table 2).

Although BVDV infections in pigs often do not cause obvious clinical features and symptoms, some of the symptoms are similar to those caused by classical swine fever virus (CSFV) infections [126], and the presence of cross-reactive antigens between BVDV and CSFV [127] can make accurate identification and diagnosis of CSFV infections challenging. The positive side is that BVDV may alter the transmission of swine fever within and between herds, when the presence of BVDV antibodies in pigs not only prevents the clinical signs of subsequent CSFV infection, but also limits or even prevents the spread of CSFV [128]. It is also worth noting that cattle can be infected with CSFV, and in one cattle testing experiment, 36 out of 40 BVDV Ab-positive samples tested positive for CSFV Ab, a positivity rate that can reach 90.0% [32].

Co-infection with multiple pathogens, including BVDV and PEDV, also exists in swine farming [34,124]. During co-infection with PEDV and BVDV, a more severe inflammatory response is often induced, causing an increase in the production of inflammatory cytokines, which may be related to exacerbating the severity of inflammatory bowel disease (IBD). Moreover, under the action of the dynamic balance mechanism of the host immune response, suppression of viral replication may occur to some extent due to the elevated cytokine levels induced by co-infection with the two viruses [124].

When BVDV and PCV are co-infected, BVDV enhances the pathogenicity of PCV-2, triggering more severe systemic PCV-2-associated disease (PCVAD), and also enhances the cellular tropism of PCV-2, leading to distinct pathological changes in renal tubular and bronchial glandular epithelium and vasculature [125], suggesting that inter-viral synergies may be achieved through host factor (e.g., cellular receptor) sharing or remodelling of inflammatory microenvironment to achieve synergy.

In addition, cases of co-infection of BVDV with one or more of the major pig-infecting pathogens, such as TGEV, PDCOV, and PKoV, have occurred in farming [33,35,129,130]. This suggests that we should be aware of false positives, co-infections and cryptic infections caused by other viral infections when testing, and consider the complexity of cross-species infections, which to a certain extent will challenge the precision of the classical swine fever eradication programme, and will also affect the safety and efficacy of some vaccines and other vaccine prevention products.

## 3. Co-Infection of BVDV with Bacteria

BVDV infection causes immunosuppression in sick animals, leading to a decrease in the body’s resistance to pathogens [47,118], which creates an ideal environment for secondary bacterial infections, during which the sick animals are not only susceptible to infection by pathogenic bacteria, but may also be invaded by opportunistic pathogens that can cause disease. This has to draw our attention to the secondary infection of bacteria (Table 3).

### 3.1. Co-Infection of BVDV with Mannheimia haemolytica

*Mannheimia haemolytica* (formerly known as Bartonella henselae) is a weakly haemolytic Gram-negative coccobacillus that is an opportunistic pathogen of cattle, sheep, and other ruminants [140], and exists as an exclusive commensal in the upper respiratory tract and nasopharynx of healthy ruminants, and is the primary bacterial agent of the Bovine Respiratory Disease Complex (BRDC) [141], which causes upper respiratory infections, pleurisy, pneumonia, and septicaemia [142,143].

In an experiment testing 90 calves, 26 calves tested positive for BVDV and 25 of these 26 tested positive for *Mannheimia haemolytica*, a co-infection rate of 96% [93]. Co-infected animals showed a biphasic serum amyloid A (SAA) response and complex cytokine interactions, which further exacerbated the infection and compromised host immunity, and more severe clinical signs were observed [132]. Severe fibrinopurulent bronchopneumonia and pleurisy occur with co-infection compared to infection alone [131]. Notably, co-infection of either cytopathogenic or non-cytopathogenic strains of BVDV with M. haemolytica causes severe respiratory disease, but the cytopathogenic strains cause more severe effects than the non-cytopathogenic strains [133]. Co-infection also resulted in reduced clearance of M. haemolytica from the lungs, leading to a significant increase in lung bacterial loads, which may be related to the fact that viral infection alters the environmental conditions of the lungs in a way that facilitates bacterial replication and reduces the host’s defences [132].

Lymphocytopenia was more pronounced in co-infections, with a decrease in the number of CD4^+^ cells and a decrease in the number of CD8^+^ and WC1^+^ cells, which showed more severe clinical symptoms [136]. In addition, the interaction of co-infections resulted in reduced antibody production to haemolytic leukotoxins and increased serum IFN-γ, IL-1β and TNF-α concentrations, suggesting that immunosuppressed viruses increase the likelihood of secondary bacterial infections and reduce the ability to produce antibodies, which may also have an impact on vaccine efficacy [134], resulting in an “immunosuppression–bacterial proliferation–inflammation imbalance”. This will also have a certain impact on the effectiveness of the vaccine, resulting in a vicious circle of “immunosuppression–bacterial proliferation–inflammation imbalance”, which further increases the difficulty of prevention and control. Meanwhile, although co-infection does not have a significant effect on the quality of internal organs, it will cause a reduction in nitrogen retention and a decrease in the proportion of ketone bodies in infected animals, and the energy allocation will be tilted towards the immune response, which will have a long-term effect on the production performance of infected animals [135].

### 3.2. Co-Infection of BVDV with Salmonella

Salmonella is a Gram-negative bacterium that includes both typhoidal and non-typhoidal Salmonella (NTS) strains and can cause a wide range of intestinal diseases in a variety of animals [144]. Cattle can be infected with a number of different serotypes of Salmonella, but the two serotypes most commonly associated with disease are Salmonella Dublin and Salmonella typhimurium, causing diarrhoea, enteritis, pneumonia, arthritis, meningitis and bacteraemia [145].

BVDV and Salmonella have been known to co-infect animals to death and transmit the disease naturally [146], and some synergistic effects have been observed in experimental calves co-infected with BVDV and Salmonella. BVDV infection induces an immunosuppressive effect that may exacerbate the effects of Salmonella infection, and concurrent infection results in more than infection with either pathogen alone [137]. Severe symptoms may be related in part to the fact that BVDV sensory suppression of lymphocyte function and weakening of mucosal barrier integrity significantly enhance Salmonella’s ability to invade, leading to more severe intestinal inflammation and systemic infection.

### 3.3. Co-Infections of BVDV with Other Bacteria

Co-infections with multiple pathogens such as BVDV and Mycobacterium bovis also occur [31] and have been shown to be associated with acute respiratory disease in calves with Pasteurella pneumophila [66].

Although co-infection with BVDV and Mycobacterium bovis increased the severity of bovine tuberculosis [147], co-infection with BVDV and Mycobacterium bovis did not result in significant shedding of pathogens or a higher probability of transmission than infection alone, and co-infection with BVDV [138] and Mycobacterium hepaticum did not significantly affect the presence of visible lesions of bovine tuberculosis caused by Mycobacterium bovis in the high-risk cohort [148].

Haemophilus influenzae infections tended to cause the development of myocarditis, pericarditis, pleurisy, polyarthritis and septicaemia, and it is noteworthy that BVDV increased the severity of Mycobacterium haemophilus-induced myocarditis [149].

Co-infection of BVDV with Chlamydia pecorum also exists in the case of natural transmission. Transient infection with BVDV leads to lymphopenia and immunosuppression, which significantly increases the susceptibility of calves to Chlamydia pecorum, allowing Chlamydia pecorum to break through the blood-brain barrier, making it more susceptible to meningoencephalitis, vasculitis and fibrin thrombosis, and the co-infections exhibited high mortality rates.

### 3.4. Co-Infections of BVDV with Atypical Bacteria

#### 3.4.1. Co-Infection of BVDV with *Mycoplasma bovis*

*Mycoplasma bovis* is a cell wall-less bacterium belonging to the group of molluscs [150], first isolated in 1961 [151], which can affect infected animals in a wide range of tissues and organs, causing respiratory diseases, mastitis, otitis media, arthritis, and reproductive disorders [152,153], and can sometimes be isolated in healthy cattle [154]. It is often mixed with different viruses and bacteria to cause BRDC. Although some teams have examined microRNAs (miRNAs) of BVDV co-infection with *Mycoplasma bovis* as biomarkers and indicators of pathogen exposure, there is still very limited in using this to assess and develop strategies to reduce bovine respiratory disease caused by their infections [155].

Co-infections of BVDV with *Mycoplasma bovis* have occurred, for example, 28 cases of mixed infections of *Mycoplasma bovis* and BVDV were detected in 49 diseased cattle tested. In vitro experiments with bovine macrophage cell lines, the presence or absence of BVDV did not significantly alter parameters such as cellular uptake and growth levels of *Mycoplasma bovis*-infected cattle [156], but persistent or primary BVDV infections make animals more susceptible to *Mycoplasma bovis* [157] and may also predispose them to chronic bacterial infections, which in turn may be implicated in the induction of chronic unresponsive pneumonia or arthritis [149,158,159]. Some studies have indicated that there may be some synergism in the co-infection of BVDV [160] and *Mycoplasma bovis*, suggesting that synergism may be achieved through indirect mechanisms (e.g., host immune remodelling) rather than direct pathogen interactions.

#### 3.4.2. Co-Infection of BVDV with Chlamydia pecorum

Chlamydia causes a wide range of diseases in its hosts (humans, domestic animals, etc.), with Chlamydia pecorum being the major ruminant pathogen, with infections causing conjunctivitis, pneumonia, bovine encephalomyelitis and reproductive disorders [161].

Co-infection of BVDV with Chlamydia pecorum also exists in the case of natural transmission. Transient infection with BVDV leads to lymphopenia and immunosuppression, which significantly increases the susceptibility of calves to Chlamydia pecorum, allowing Chlamydia pecorum to break through the blood-brain barrier, making it more susceptible to meningoencephalitis, vasculitis and fibrin thrombosis, and the co-infections exhibited high mortality rates [139].

## 4. Co-Infection of BVDV with Other Pathogens

BVDV exists not only as a co-infection with viruses and bacteria, but also as a mixed infection with other pathogens, such as Neospora, causing more serious harm.

### Co-Infection of BVDV with Neospora caninum

*Neospora caninum* is an exclusively intracellular parasitic protozoan-like parasite [162] that infects dogs, cattle and other animals. Among them, Neosporosis in cattle caused by infection of cattle can result in abortion, foetuses may be dead in utero, absorbed, mummified, autolysed, stillborn, clinically symptomatic at live birth, or clinically normal at birth but chronically infected, and calves may show neurological and other signs [163]. Neospora-induced abortions usually occur in early, mid-, or even throughout pregnancy [64], and BVDV often also causes immunosuppression and early embryonic death [51].

Co-infection with various pathogens such as Neospora canis and BVDV exists and occurs naturally [64,164,165,166,167], and a previous investigative study suggested a possible association with abortion [164], but subsequent studies have not clearly found that BVDV infection affects the risk of abortion in Neospora canis [165]. In co-infections, there is a correlation between *Neospora caninum* positivity and BVDV infection [64], and some studies have found a possible interaction between *Neospora caninum* and BVDV. It is hypothesised that immunosuppression by BVDV may be a possible contributing factor to outbreaks of abortions associated with *Neospora caninum* [164], possibly due to a weakening of the immune control of *Neospora caninum* by BVDV, which increases the risk of colonization and proliferation of the parasite in the placental tissues.

## 5. Conclusions

**Figure 1:**
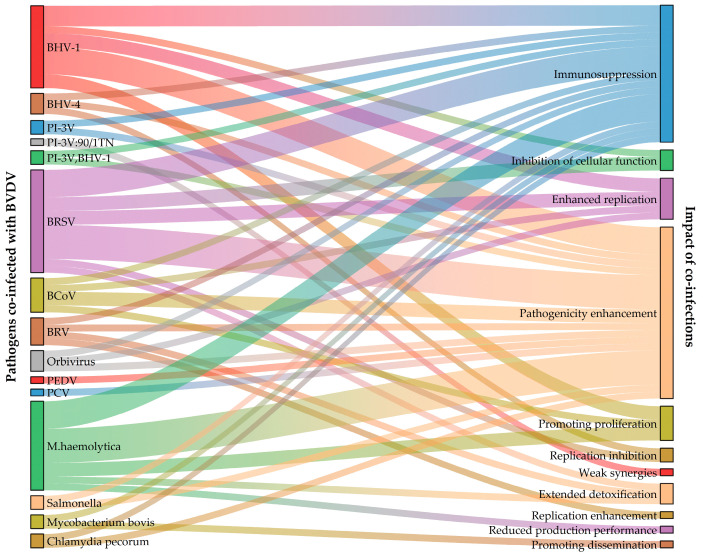
Synergistic effects of co-infection of BVDV with multiple pathogens.

In summary, simple BVDV infection generally causes gastrointestinal disorders, miscarriage, mucosal disease, and immunosuppression, although different genotypes cause different major clinical symptoms, and there are cases of different genes co-infecting the same individual. The most noteworthy is the central role of immunosuppression, which makes animals more susceptible to secondary infections with other pathogens, leading to a diversity of pathogen interactions. Co-infection of BVDV with other pathogens not only enhances the pathogenicity and causes superimposition of symptoms, which results in more severe clinical symptoms and diseases, but also causes a prolonged course of the disease and longer detoxification time, and other conditions (Figure 1).

Through this study, it is systematically known that BVDV co-infection complicates the host pathological process through pathogen synergism, contributes to the superimposition of symptoms or pathologies of multiple infections, and even induces novel pathologies due to pathogen interactions, and exhibits a more severe clinical response in general, significantly destroys the body’s innate immune barriers (mucous membranes, etc.), exacerbates the failure of the body’s first line of immune defence, and thus facilitates the invasion of secondary pathogens. In severe cases, the process of death can be accelerated by multi-system damage. Not only does BVDV cause immunosuppression, but some pathogens also cause a certain degree of immunosuppression, exacerbating the impact on the immune system, weakening the control of the pathogen, and even jointly inhibiting the clearance of the pathogen, forming a vicious circle leading to the collapse of immune defences, and encouraging further multiplication of the pathogen in the body and systemic spread of the pathogen. Compared with single infections, co-infected host target organs may have significantly higher pathogen titres and promote the excretion of one or more pathogens from the body, resulting in prolonged excretion of pathogens, which greatly enhances the speed and extent of pathogen transmission. If the synergistic effect of co-infection develops from the “cascade effect” at the individual level to the group, forming a vicious cycle of “disease exacerbation-immune imbalance-pathogen proliferation-spreading and dissemination”, it will certainly pose a serious challenge to the prevention and control system of animal diseases.

It is worth noting that BVDV-induced suppression of the immune function of the diseased animal reduces the host animal’s immunity and makes it more susceptible to the invasion of other susceptible pathogens, and even to infections by opportunistic pathogenic microorganisms. Following the onset of co-infection, there is a decline in animal performance and an increase in treatment costs, and a number of synergistic interactions between pathogens and BVDVs that work together to promote the onset and exacerbation of clinical signs in infected animals, resulting in a more severe and complex disease that leads to higher culling rates and mortality.

Co-infection can also present difficulties in the accurate diagnosis of disease, as there may be similarities in clinical signs between pathogens. The detection of pathogens can also be affected by inter-pathogen influences, leading to misdiagnosis or false positives. Meanwhile, attention should be paid to the detection and control challenges posed by cross-species transmission of BVDV. In addition, special attention needs to be paid to the potential risks of bovine vaccine biologics, noting that the use of bovine-derived cell lines to culture the virus during vaccine production may present a risk of BVDV contamination, and that contaminated strains may be transmitted to the herd through vaccination. This can cause considerable difficulties in pathogen identification, treatment, vaccine selection and management measures, resulting in huge economic losses and prevention and control challenges for the cattle industry.

Therefore, high priority should be given to the prevention and control of BVDV, and effective measures should be taken to reduce the risk of co-infection with other pathogens in actual production, and further attention should be paid to vaccine safety, diagnostic methods for co-infection, interactions, effects on the host and prevention. The above review may provide some help and ideas for diagnostic optimisation, vaccine and therapeutic innovation, prevention and control of diseases associated with BVDV co-infection. Some of the mechanisms of co-infection are still under-researched, and most of the synergistic mechanisms are still in the description of the phenomenon. The animal models previously studied have some limitations, and most of the existing experiments are based on in vitro cells or calves, and it is necessary to construct an adult cattle model that is closer to the natural infection to simulate the situation in natural production.

In the future, it is worthwhile to pay attention to the synergistic pathogenic mechanism and immune regulation of BVDV co-infections, which will have a certain impact on the effectiveness of vaccine prevention and control. In this regard, it is necessary to break through the traditional thinking of the prevention and control of a single pathogen, and to explore the interactions among the three parties, parties, vaccine, pathogen, and host, in order to achieve the efficient prevention and control of co-infections.

## Figures and Tables

**Table 1:** Co-infection of BVDV with viruses.

Coinfections	Protocols	Selected Cells or Bovine	Observations	References
BVDV BHV	BVDV BHV-1: Colorado natural infection In vivo	Cattle: Calves and Adult cattle	- BVDV inhibits viral clearance mechanisms in the lungs and prolongs BHV1 replication in the lungs - BVDV enhances the pathogenicity of BHV-1, leading to more severe respiratory tract damage	[36,37]
	BVDV-1 BHV-1.1 BVDV then BHV In vivo	Cattle: 8-to 9-month-old calves (without BVDV/BHV-1 antibodies and antigens)	- Co-infections lead to more severe clinical symptoms and pathological changes - Co-infection enhances the replication and transmission of both viruses in all tissues and organs, creating a two-way synergistic effect - BVDV impairs early host immune control of BHV-1 by inhibiting anti-inflammatory factors and key Th1 cytokines	[38,39,40,41,42,43]
	BVDV BHV-1 BVDV then BHV-1 In vivo	Cattle: 4-to 6-month-old calves	- Co-infection leads to more severe acute respiratory disease - Co-infection enhances BHV-1 pathogenicity	[44]
	BVDV BHV-1 PI-3V natural infection In vivo	Cattle: 1-week-to 12-month-old calves	- BVDV infection leads to lymphocyte depletion and significant suppression of the host immune system, contributing to secondary infection with BHV-1 and PI-3V - Co-infections with the most severe clinical symptoms (high fever, diarrhoea, respiratory symptoms) and high mortality rates	[45]
	BVDV: NY-1; TVM2 BHV-4: 81/16TV BHV-1: 90/180TN BVDV then BHV-4, BHV-1 In vivo	Cattle: 30-to 40-day-old calves (without antibodies to associated viruses)	- Sequential infection with BHV-4 reactivated BVDV (especially the CP strain), leading to more severe immunosuppression and clinical symptoms - BHV-1 may enhance BVDV pathogenicity and promote virus spread in non-respiratory tissues	[46]
	Simultaneous infection In vivo		- Concurrent infections lead to severe clinical symptoms, including high fever, diarrhoea and leucopenia	[46]
	BVDV-1 BHV-4: 10/154 BVDV then BHV-4 In vitro	Cells: Endometrial cells (Natural infection with NCP-type BVDV)	- BVDV delays BHV-4 gB gene expression and inhibits replication efficiency - BVDV infection significantly reduces BHV-4 viral load in BEC	[47]
BVDV PI-3V	BVDV PI-3V Natural infection In vivo	Cattle: 1-week-to 12-month-old calves	- BVDV infection leads to lymphocyte depletion and significant suppression of the host immune system, contributing to secondary infection with PI-3V - Co-infections exacerbate respiratory symptoms	[45]
	BVDV: NY-1; TVM PI-3V: 90/1TN Simultaneous infection In vivo	Cattle: 30- to 40-day-old calves (without antibodies to associated viruses)	- Weak synergistic effect, triggering only mild respiratory symptoms and transient leukopenia	[46]
BVDV BRSV	BVDV: NY-1 BRSV: 165 Simultaneous infection In vivo	Cattle: Calves	- Co-infections significantly increased BRSV pathogenicity with more severe respiratory symptoms and more extensive lung lesions	[48]
	BVDV: 187/92 BRSV: 504/93 Simultaneous infection In vivo	Cattle: 14-to 17-week-old calves (without antibodies to BVDV, IBR and BLV)	- BVDV induces interferon release and inhibits BRSV replication - Co-infections present with delayed antibody response, prolonged viral excretion and more persistent fever	[49]
	BVDV: NY-1 BRSV: 236-652 Simultaneous infection In vivo	Cattle: 9-to 12-month-old calves	- Co-infections inhibit lymphocyte proliferation and function and exacerbate inflammatory responses - Exacerbation of BRSV-infected respiratory and gastrointestinal tract lesions leading to more severe disease manifestations	[50]
	BVDV BRSV Simultaneous infection In vivo	Cattle: 9- to 12-month-old calves	- Co-infections present with more severe fever, diarrhoea and leucopenia - Co-infections significantly exacerbate respiratory (e.g., lung lesions) and digestive diseases in calves - Successful isolation of BRSV in co-infected lung tissue only	[51]
	BVDV-2-wt BRSV: 236-652 BVDV then BRSV In vitro	Cell: Bovine turbinate cells; MDBK cells	- Inhibition of IFN-1 by co-infection leads to a weakened host immune response and enhanced BRSV replication - Exacerbation of clinical symptoms and disease severity	[52]
	BVDV: ncp BRSV: 375 Simultaneous infection In vitro	Cell: Bovine alveolar macrophage	- Co-infection inhibits Fc receptor expression, phagosome-lysosome fusion, superoxide anion (O₂-) production and chemokine secretion in bovine alveolar macrophages - Co-infection enhances BRSV replication	[53]
BVDV BCoV	BVDV-2a: RS886 BCoV: OK 1776 BVDV then BCoV In vivo	Cattle: 2-to 5-week-old calves	- Co-infection can enhance the nasal detachment of BVDV - Co-infection enhances the replication and pathogenicity of BCoV, leading to more severe lung lesions and facilitating the spread of the virus.	[54]
	BCoV then BVDV In vivo		- Synergistic exacerbation of respiratory disease	[54]
	BVDV-1 BCoV BVDV then BCoV In vivo	Cattle: 59-to 165-day-old calves	- Co-infections present with more severe clinical symptoms	[55]
BVDV BRV	BVDV: NY-1c BRV: NS-1 BVDV then BRV In vivo	Cattle: 1-day-old calves (GF)	- Co-infections have a synergistic effect on intestinal damage, leading to more severe enteritis - BVDV enhances BRV pathogenicity and prolongs viral excretion of BRV	[56]
	Simultaneous infection In vivo		- Coinfection with BRV enhances BVDV replication	[56]
BVDV Orbivirus	BVDV: ncp (Positive END phenomenon) Orbivirus BVDV then Orbivirus In vitro	Cells: Bovine testicle cells	- Co-infection blocks the host interferon response and weakens antiviral defences, thereby promoting circovirus proliferation and inducing CPE	[57]

**Table 2:** BVDV and other co-infections associated with porcine viruses.

Coinfections	Protocols	Selected Cells or Porcine	Observations	References
BVDV PEDV	BVDV-2: SH-28 PEDV: JS-2/2014 Simultaneous infection In vitro	Cells: Porcine kidney (PK15) cells	- Co-infections activate the IBD pathway and NF-κB signalling pathway, promoting the expression of inflammatory factors and triggering a stronger intestinal inflammatory response	[124]
BVDV PCV	BVDV-1 PCV-2 Simultaneous infection In vivo	Porcine: 24-day-old piglets (GF)	- Co-infection may exacerbate PCV-2 pathogenicity under certain conditions (e.g., immune stimulation)	[125]

**Table 3:** Co-infection of BVDV with bacteria.

Coinfections	Protocols	Selected Cells or Bovine	Observations	References
BVDV M. haemolytica	BVDV: 72 M. haemolytica-1 BVDV then M. haemolytica In vivo	Cattle: 4- to 6-month-old calves	- Reducing bacterial clearance and promoting lung colonisation and spread of M. haemolytica - Co-infection presenting as severe fibrinopurulent pneumonia and pleurisy	[131]
	BVDV 1 M. haemolytica: Ab 35 BVDV then M. haemolytica In vivo	Cattle: 9-to 18-week-old calves	- BVDV weakens host defences, promotes Mh colonisation, delays bacterial clearance and prolongs the disease process - Synergistic effects lead to a more severe clinical course and a more prolonged immune response	[132]
	BVDV: 2724; 72 M. haemolytica-1 BVDV then M. haemolytica In vivo	Cattle: Calves	- Co-infections produce a more severe synergistic effect with more severe respiratory symptoms and lung lesions - Co-infections reduce bacterial clearance from the lungs and promote transmission	[133]
	BVDV-1b M. haemolytica BVDV then M. haemolytica In vivo	Cattle: Crossbred beef cattle	- BVDV enhances the pathogenicity of M. haemolytica - Co-infections present with more severe clinical symptoms, stronger inflammatory response	[134]
	BVDV-1b M. haemolytica-1 BVDV then M. haemolytica In vivo	Cattle: Angus crossbred steers	- Co-infections have a synergistic negative effect on beef cattle in the form of growth inhibition, disturbances in nitrogen metabolism and changes in internal organ quality	[135]
	BVDV M. haemolytica BVDV then M. haemolytica In vivo	Cattle: 12-week-old calves	- Co-infection synergistically suppresses cellular immunity to exacerbate the severity of M. haemolytica infection - Co-infections lead to more persistent immunosuppression and more severe clinical symptoms	[136]
BVDV Salmonella	BVDV Salmonella Co-infection In vivo	Cattle: Calves	- Pathogenicity and impact of BVDV in exacerbating Salmonella infections - Co-infection leads to more severe clinical illness	[137]
BVDV Mycobacterium bovis	BVDV: 11249 Mycobacterium bovis: AF 2122/97 Mycobacterium bovis then BVDV In vivo	Cattle: Calves	- Co-infection reduces the accuracy of diagnostic tests for bovine tuberculosis - Co-infection affects frequency of excretion or lesion dynamics indirectly facilitating transmission	[138]
BVDV Chlamydia pecorum	BVDV: ncp Chlamydia pecorum Natural infection In vivo	Cattle: 12-to 18-week-old calves	- Co-infections cause meningoencephalitis, vasculitis and fibrin thrombosis	[139]

## Abbreviations

The following abbreviations are used in this manuscript:

BVDV	Bovine viral diarrhoea virus
BVD-MD	Bovine viral diarrhea/mucocutaneous disease
BRDC	Bovine respiratory disease complex
BHV	Bovine herpesvirus
PI-3V	Parainfluenza virus type 3
BRSV	Bovine respiratory syncytial virus
BCoV	Bovine coronavirus
BRV	Bovine rotavirus
PEDV	Porcine epidemic diarrhea virus
PDCOV	Porcine delta coronavirus
PKoV	Porcine kobuvirus
PASTV	Porcine stellate virus
TGEV	Transmissible gastroenteritis virus
IBR	Infectious bovine rhinotracheitis
BPV	bovine polyomavirus
PPRV	Peste des petits ruminants virus
PCPV	Pseudocowpox virus
BLV	Bovine Leukemia Virus
BAV	Bovine adenovirus
FMDV	Foot-and-mouth disease virus
CSFV	Classical swine fever virus
PCV	Porcine circovirus
MH	*Mannheimia haemolytica*
